# Insights into the Mechanisms Driving the Dynamics of Antibiotic Resistance Genes During Pig Manure Composting

**DOI:** 10.3390/toxics14070636

**Published:** 2026-07-21

**Authors:** Xun Pan, Rukun Cao, Mengxue Ge, Mengqi Dong, Weiwei Ben

**Affiliations:** 1 Solid Waste and Chemicals Management Center, Ministry of Ecology and Environment of the People’s Republic of China, Beijing 100029, China; 2 Research Center for Eco-Environmental Sciences, University of Chinese Academy of Sciences, Chinese Academy of Sciences, 18 Shuang-qing Road, Beijing 100085, China

**Keywords:** antibiotic resistance genes (ARGs), composting, thermophilic stage, mobile genetic element (MGE), bacterial community

## Abstract

As a widely adopted approach for the resource utilization of pig manure, the ability of composting to reduce risk pollutants such as antibiotic resistance genes (ARGs) has gained significant attention. Temperature plays a pivotal role in determining the effectiveness of composting, with the maximum composting temperature and duration of high temperature being key indicators of compost quality. This study investigated the influences of adjusting the thermophilic stage on the dynamics of ARGs during pig manure composting. The results revealed that the adjustment strategies of thermophilic stage controls (i.e., prolonging the duration of the thermophilic stage or raising the maximum temperature) slightly promoted the absolute abundance of total ARGs by 0.72–0.99 logs, whereas their relative abundance was notably reduced by 49.7~64.1%. Some ARGs (i.e., *tet*W, *tet*O, *tet*M, *fex*A, *fex*B, *erm*A, and *erm*B) could be effectively removed by the composting, whereas *sul*I, *sul*II, *aad*A, and *tet*L enriched the horizontal gene transfer and diversified the potential bacterial hosts. The variations of ARG profiles and the succession of bacterial communities could be divided into two stages, which coincided with the organic carbon (OC) content (82%). The nutrient factors, especially the OC content, were strongly relative to several ARGs, implying that the organic nutrient could be an important driving force in shaping ARG distribution, potentially by influencing bacterial community succession. Three potential opportunistic pathogens (*Mycobacterium*, *Bordetella* and *Bacillus*) exhibited positive correlations with enriched ARGs, highlighting the potential risks of the dissemination of antibiotic-resistant pathogens though the application of compost products.

## 1. Introduction

As a highly efficient approach for managing livestock waste, composting converts pig manure into a manure-based organic fertilizer that holds promise as a substitute for synthetic fertilizers. However, the waste products usually contain abundant antibiotic resistance genes (ARGs), which poses a substantial health concern [1,2]. For pig manure, ARGs are frequently detected due to the large-scale use of antibiotics (e.g., 48,400 tons in 2013) in the Chinese pig industry [3,4]. Through horizontal gene transfer (HGT), pathogenic bacteria could acquire ARGs, thereby gaining resistance to clinically relevant antibiotics, and threatening human health [5,6]. Current studies demonstrate various fates for different ARGs, some of which decreased whereas others increased in manure composting, and different studies observed inconsistent variations in the same ARGs for different composting conditions [7,8,9,10]. This inconsistency underscores the need for further investigation into the key factors governing ARG fate, as well as for the effective strategies aimed at their elimination during composting.

Temperature is the most direct and sensitive indicator to evaluate bacterial activities and maturity in composting [11], and is demanded by the relevant standards or guidelines in various countries [12,13]. The composing process is typically segmented into three phases based on temperature dynamics: (1) mesophilic stage, temperature rises to around 45 °C and mesophilic bacteria are dominant; (2) thermophilic stage, temperature remains above 50 °C and thermophilic bacteria are dominant; (3) cooling and maturation stage, temperature gradually returns to ambient and mesophilic bacteria become dominant species again. It indicates a close relationship between pile temperature and bacterial community, where the bacterial activity generates heat to change the pile temperature and the variation in temperature promotes the succession of bacterial communities. Temperature is recognized as a critical determinant of ARG profiles. For instance, Li et al. [14] identified temperature as the most influential environmental factor shaping ARG composition during composting, with the majority of ARG removal occurring in the thermophilic phase. Liu et al. [15] observed that the ARGs and mobile genetic elements (MGEs) could recover along with the compositing and even excessed the initial abundances. Our previous study indicated that both ARG profiles and bacterial community succession were distinctly demarcated by the mesophilic–thermophilic and cooling–maturation boundaries defined by pile temperature, suggesting that manipulation of the thermophilic stage may represent a viable approach for ARG attenuation [16]. Liao et al. [17] achieved hyperthermophilic and conventional composting by forced aeration and natural ventilation with turning, respectively, and reported that hyperthermophilic composting accelerated the removal of ARGs and MGEs. Qian et al. [18] achieved three temperature regimes in composting using a simulated 500 mL bottle experiment in a biochemical incubator, and reported that the continuous thermophilic composting performed significantly better at reducing ARGs and integrons. However, the mechanism that influences temperature in the ARG profile in composting is still ambiguous, and studies of the thermophilic stage control to remove ARGs in composting are still insufficient.

In the present study, taking the spontaneous thermogenic composting by heat insulation as control treatment, the controls for the thermophilic stage (i.e., prolonging the duration and raising the temperature) by heating with a water bath were used in pig manure composting. The dynamics of ARGs and MGEs, the bacterial community succession, and the main physicochemical parameters were determined. The objectives of this study are to elucidate the effects of thermophilic-stage regulation on the ARG profile and bacterial community structure, and to assess the practical feasibility of removing ARGs through the controls of the thermophilic stage.

## 2. Materials and Methods

### 2.1. Experimental Condition

Pig manure samples were obtained from a pig farm in Beijing, China. Wheat straw was used as a supplemental material in the composting. The pig manure and wheat straw had the following properties, respectively: moisture content (MC) of 69.2% and 6.1%; organic carbon (OC) of 82.5% and 89.1%; total carbon (TC) of 39.6% and 41.9%; total nitrogen (TN) of 4.24% and 1.07%; and pH of 5.2 and 5.3. The compost mixture was prepared by mixing pig manure with wheat straw at a fresh weight ratio of 1:1.2 (the TC/TN ratio of about 25), and then adjusted the MC to 55%. Composting was conducted in four identical cylindrical stainless steel reactors (H 60 cm, Ø 35 cm) with a sleeve (thickness 10 cm) outside. The sleeve was used for water bath heating in the thermophilic stage, and was filled with heat insulating materials at other stages. Four experiment treatments were named according to the control of thermophilic stage: (1) the CK group with the spontaneous thermogenic method, the reactor was wrapped with insulating material for heat preservation throughout composting, and the thermophilic stage (T > 50 °C) lasted 9.5 d in this study; (2) the 55(9.5) group, the reactor was heated using a water bath at 55 °C for 9.5 d when the compost pile entered the thermophilic stage; (3) the 55(19) group for prolonging the duration of thermophilic stage, the reactor was heated using a water bath at 55 °C for 19 d when the pile entered the thermophilic stage; (4) the 70(9.5) group to raise the temperature of the thermophilic stage, the reactor was heated using a water bath at 70 °C for 9.5 d when the pile entered the thermophilic stage. An air compressor in the bottom of the reactor aerated for 0.16 m^3^ h^−1^ to provide oxygen to the composting pile. A pulse-aeration strategy was implemented, consisting of 90-s on-periods at an airflow of 0.24 m^3^·h^−1^, succeeded by 10 min of interruption. Water was sprayed appropriately onto the surface of the compost piles each day to prevent superficial drying.

The ore temperature of each compost pile was monitored in real time by a sensor. Sampling was performed on the designated days and the physicochemical parameters of the compost samples were analyzed according to the Standard Methods for the Examination of Water and Wastewater (APHA, 2005) [19]. Specifically, MC and OC were quantified by oven-drying fresh specimens at 105 °C, followed by combustion in a muffle furnace at 600 °C. The pH was measured with a pH meter (FE20, Mettler Toledo, Greifensee, Switzerland) in 1:10 (*w*/*v*) fresh sample-to-deionized water suspensions. Samples were extracted with 1 M KCl at a ratio of 1:20 (*w*/*v*), and the resulting extracts were analyzed for NO_3_^−^-N and NH_3_-N using a spectrophotometer via HACH methods 10020 and 10031, respectively. Freeze-dried samples were analyzed for TC and TN an elemental analyzer (Elementar, Langenselbold, Germany). A portion of the freeze-dried sample was stored for DNA extraction.

### 2.2. DNA Extraction and Quantitative PCR (qPCR)

Genomic DNA was extracted in triplicate freeze-dried sample using a FastDNA Spin Kit for soil (MP-bio, Solon, OH, USA) according to the manufacturer’s protocol, and stored at −20 °C until use. DNA concentration and quality were evaluated with a Genequat 1300 spectrophotometer (GE Healthcare, Cardiff, UK) and agarose gel electrophoresis, respectively.

SYBR Green real-time qPCR was applied to quantify 37 target genes, specifically tetracycline resistance genes (TRGs, *tet*A, *tet*A/P, *tet*C, *tet*H, *tet*L, *tet*M, *tet*O, *tet*Q, *tet*W, and *tet*X), sulfonamide and trimethoprim resistance genes (STRGs, *sul*I, *sul*II, *dfr*A1, and *dfr*A7), macrolide, lincosamide and streptogramin B resistance genes (MLSBRGs, *erm*A, *erm*B, *erm*C, *erm*F, and *mef*A/E), β-lactam resistance genes (β-LRGs, *bla*CXM, *bla*OXA-1, and *bla*TEM), quinolone resistance genes (QRGs, *aac*(6′)-Ib-cr, *gyr*A, and *par*C), chloramphenicol resistance genes (CRGs, *cfr*, *cml*A, *fex*A, *fex*B, and floR), aminoglycoside resistance genes (AmRGs, *aac*(3)-II, *str*A, *str*B, *aad*A, and *aad*B), polymyxin resistance gene (PRG), and MGEs (*int*I1, *int*I2, Tn916/1545, and *Inc*Q *ori*T). Plasmid standards carrying each target genes were diluted ten-fold to establish standard curves from 10^8^ to 10^1^ copies μL^−1^. All reactions were run in triplicate on an ABI Prism 7300 platform (Applied Biosystems, Foster City, CA, USA); standard curves with R^2^ > 0.99 and amplification efficiency within 90–110% were deemed valid. The primers, annealing temperatures, amplification efficiencies, reaction mixtures, and thermal profiles are listed in Table S1. The absolute abundances of genes were given as copies g^−1^ dry weight, with relative abundances normalized to 16S rRNA copy numbers.

### 2.3. High-Throughput Sequencing and Bioinformatics Analysis

Equal volumes of the triplicate DNA extracts were combined and used as a template for amplifying the 16S rRNA V3–V4 region with primers 338F/806R. Amplicon libraries were constructed and sequenced (Illumina MiSeq, 2 × 300 bp) with Majorbio Bio-Pharm Technology (Shanghai, China). The primers were (50-ACTCCTACGGGAGGCAGCAG-30) and 806R (50-GGACTACHVGGGTWTCTAAT-30). Raw reads are accessible at NCBI SRA under SRP257858. Read processing involved demultiplexing, quality filtering (Trimmomatic, v0.39), and merging (FLASH, v1.2.11) [20]. Operational taxonomic units (OTUs) at 97% similarity were clustered with UPARSE (v.7.1), and chimeras were removed with UCHIME (v2.13) [21]. Each sample was rarefied to 18,493 reads based on the minimum library size (27,831 reads). Taxonomic annotation was performed via RDP Classifier (v2.13, 70% confidence) against the Silva SSU123 database.

### 2.4. Statistical Analysis

Two different grouping schemes were applied to the samples for molecular biology analysis (Table 1): (1) By control of the thermophilic stage (CK, 55(9.5), 55(19) and 70(9.5)); (2) By temperature variation stage (raw, mesophilic, thermophilic, transition, cooling, and maturation).

Graphical outputs were generated using OriginPro 8.0 (OriginLab Corp., Northampton, MA, USA). Ordination analyses, specifically principal component analysis (PCA) and redundancy analysis (RDA), were conducted with Canoco 4.5 for Windows. Bacterial community composition patterns were displayed as heat maps produced with HemI 1.0 [22]. Spearman’s rank-order correlations were calculated with SPSS 18.0 to examine associations among ARGs, MGEs, physicochemical variables, and microbial communities (significance level: *p* < 0.05), and the resulting co-occurrence network was rendered using Cytoscape v. 3.6.1 [23].

## 3. Results and Discussion

### 3.1. Temporal Variations in Physicochemical Factors

**Figure 1:**
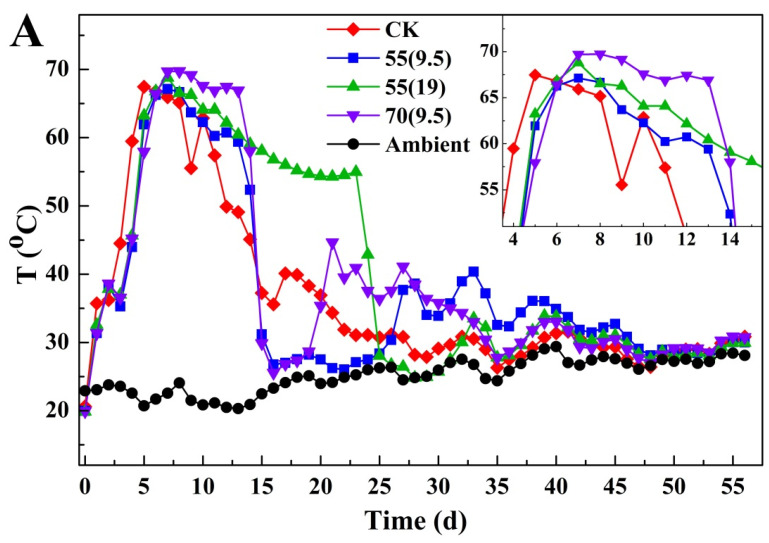

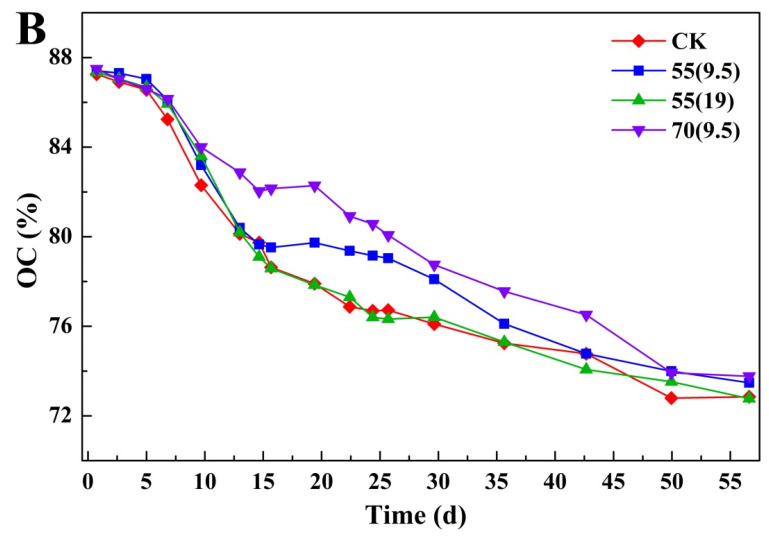
Temporal variations in core temperature (**A**) and organic content (**B**) of each compost mixture during composting. CK, 55(9.5), 55(19), and 70(9.5) were the experiment groups according to the control of the thermophilic stage. Organic content (OC) was calculated by dry weight.

The temperature variations of each pile showed significant differences due to the control of the thermophilic stage (Figure 1A). The CK, a spontaneous thermal treatment with heat insulation, entered the thermophilic stage (T > 50 °C) on day 5, which lasted for 9.5 d, and then cooled down gradually. Samples 55(9.5), 55(19), and 55(9.5) entered the thermophilic stage and were heated by a water bath on day 6. Thereafter, 55(9.5) lasted in the thermophilic stage for 9.5 d, which was similar to CK; 55(19) lasted in the thermophilic stage for 19 d, but the pile temperature was lower than the water bath (55 °C) at the later thermophilic stage (day 19–24), which showed that the pile can not maintain a high temperature spontaneously; 70(9.5) lasted in the thermophilic stage for 9.5 d with a higher temperature (>65 °C), while the pile temperature was higher than the water bath (70 °C) only on one day (day 7) during the thermophilic stage, which showed that the heat of the pile mainly came from the water bath. After water bath heating, the temperature of these three piles cooled down sharply, and rose again differently until they were close to ambient. According to the compost quality criteria GB 7959–2012, all four treatments reached the recommended threshold of sustaining ≥ 50 °C for 10 days [12].

Although the OC values reduced from 87% to around 73% in all four treatments during composting, the variation processes showed obvious differences in the control of the thermophilic stage (Figure 1B). The OC values of CK and 55(19) were similar in that they continuously declined throughout composting, especially at the thermophilic stage. For 55(9.5), the OC value was similar to CK and 55(19) before finishing the water bath heating (day 14), then remained at around 79% for 11 d with the sudden drop of temperature, and finally declined gradually at the cooling–maturation stage. For 70(9.5), the OC value declined slower than in the other groups at the later thermophilic stage (day 9 to 14), then remained at round 82% for 5 d, and finally declined gradually. Hence, the control of thermophilic stage would disturb the changes in pile temperature and OC during pig manure composting. Moreover, the pH values were slightly alkaline, the C/N ratios were below 20, the ratios of final to initial C/N were less than 0.6, and the dissolved NH_3_–N concentrations were lower than NO_3_–N after composting (Figure S1), indicating that all compost products were mature.

### 3.2. Dynamics of ARGs and MGEs During Composting

**Figure 2:**
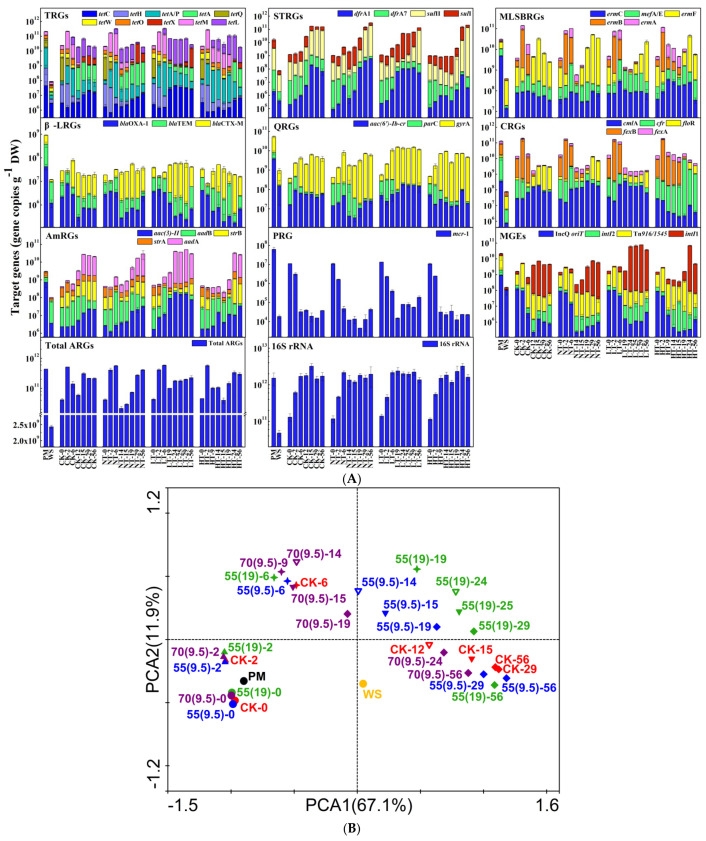
Profiles and dynamics of ARGs and MGEs during composting. (**A**) Absolute abundance of ARGs, MGEs, total ARGs, and 16S rRNA. (**B**) Principal component analysis (PCA) ordination illustrating the temporal shifts in ARG composition profiles. Black, golden, red, blue, green, and purple symbols represent PM, WS, CK, 55(9.5), 55(19), and 70(9.5), respectively. Symbols ●, ▲, 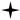, ▽, ▼, and ◆ represent raw, mesophilic, thermophilic, transition, cooling, and maturation samples, respectively.

The 16S rRNA copy numbers—an indicator of microbial biomass—increased from 10^11^ to 10^12^ copies g^−1^ DW during the initial composting phase, after which they remained stable at around 10^12^ copies g^−1^ DW through to the end of composting (Figure 2A). The ARG profiles of the raw composting mixtures were largely attributable to PM, as the absolute abundance of total ARGs in PM exceeded that in WS by 2.42 logs, and the ARG composition of PM closely resembled that of the raw samples.

As shown in Figure 2A and Figure S2, similar temporal dynamics were observed for the absolute and relative abundances of total ARGs: enriched at the mesophilic stage, then obviously declined at the later thermophilic or transition stage, and finally rebounded at the cooling–maturation stage. At the end of the composting process, the absolute abundances of total ARGs in CK, 55(9.5), 55(19), and 70(9.5) were enriched by 0.71, 0.99, 0.72, and 0.81 logs, respectively, while relative abundances declined by 66.2%, 64.1%, 49.7%, and 55.9%, respectively. These results suggested that: (1) non-ARG carrying bacteria proliferated during composting, because the fitness costs of harboring ARGs weaken the competitiveness of resistant bacteria [24]; and (2) the controls of the thermophilic stage were ineffective to promote the removal of total ARGs and even exacerbated the enrichment of total ARG absolute abundance compared with CK. Zhang et al. [9] and Bao et al. [25] observed that total ARGs rebounded at the cooling–maturation stage in pig manure composting, Wang et al. [8] observed that total ARGs were enriched after pig manure composting, and Liao et al. [26] reported that the absolute abundance of total ARGs was enriched but relative abundance declined in food waste composting. These previous results further supported our point that ARGs had higher environmental risks in traditional methods and that further measures should be taken to reduce ARGs in pig manure composting.

As shown in Figure 2A, the absolute abundances of CRGs and PRG significantly decreased by 0.37–1.97 and 1.86–2.67 logs, respectively, after composting. However, STRGs and AmRGs both increased in absolute abundance during composting, from 10^9^ to 10^11^ and from 10^8^ to 10^10^ copies g^−1^ DW, respectively, likely owing to their frequent linkage with different kinds MGEs that facilitate HGT [27,28]. QRGs also rose from 10^8^ to 10^10^ copies g^−1^ DW in absolute terms, but their relative abundance held steady at 10^−3^ throughout composting. TRGs and β-LRGs fluctuated at 10^10^ and 10^7^ copies g^−1^ DW in absolute abundance, respectively, but the relative abundances significantly declined after composting. For each ARG, *tet*W, *tet*O, *tet*M, *fex*A, *fex*B, *erm*A, and *erm*B were the major decreased contributors, and the absolute abundances declined from 10^9^–10^11^ to 10^6^–10^9^ copies g^−1^ DW during composting. Moreover, *par*C and *mcr*-1 significantly decreased from 10^8^ to 10^6^ copies g^−1^ DW and from 10^7^ to 10^4^ copies g^−1^ DW, respectively. Instead, the absolute abundances of *sul*I, *sul*II, *aad*A, and *tet*L were enriched from 10^7^–10^9^ to 10^10^–10^11^ copies g^−1^ during composting, which were the major increased contributors. Moreover, *tet*A, *tet*X, and *dfr*A1 were significantly enriched by 1.44–2.19, 1.53–2.87, and 1.50–3.82 logs, respectively.

Figure 2A demonstrated that dynamics of ARG absolute abundance were clearly divided into earlier and later composting periods for all four treatments. MLSBRGs were mainly represented by e*rm*A, *erm*B, and *erm*F in the earlier composting period; however, *erm*F became the unique dominant of MLSBRGs in the later composting period owing to the significant reduction in *erm*A and *erm*B. Similarly, *gyr*A and *par*C were the major species of QRGs with similar abundance in raw samples; however, *gyr*A became the unique dominant of QRGs in the later composting period because of the significant reduction in *par*C and the enrichment in *gyr*A. The PCA ordination (Figure 2B) clearly distinguished the ARG profiles of each group across different composting stages, and samples were distributed in a clockwise direction in the graph. Samples from the earlier and later composting period roughly clustered in the left and right part of graph, respectively. Of note, thermophilic sample 55(19)-19 was clustered with other cooling and maturation samples, while transition sample 70(9.4)-14 and cooling sample 70(9.4)-15 were clustered with other thermophilic samples. These results indicated that: (1) prolonging the thermophilic stage in the 55(19) group did not obstruct the ARG profile from entering into the later period when the organic nutrient was insufficient on days 19–24, even though the pile was heated with a higher temperature, and (2) raising the temperature of the thermophilic stage in the 70(9.5) group suspended the ARG profile from entering into the later period, even though the pile stopped being heated on days 14–15. This might be due to the fact that the excessive temperature inhibited microbial activity and consumption of organic nutrients. In short, the controls of the thermophilic stage made the earlier and later period of the ARG profile different from the mesophilic–thermophilic and cooling–maturation stage due to the pile temperature.

MGEs (e.g., integrons, transposons, and plasmids) mediate HGT and enable bacterial acquisition of ARGs in the environment [29,30]. After composting, the total MGEs and absolute abundances of CK, 55(9.5), 55(19), and 70(9.5) rose by 0.45, 0.62, 1.34, and 0.51 logs, respectively (Figure 2A), with thermophilic-stage controls promoting greater ARG enrichment than CK. Similar to the ARG trends, MGEs in all treatments split into early and late phases. Compositionally, *Tn916/1545* dominated early, whereas *intI*1 prevailed later. Specifically, *intI*1 increased from 10^8^ to 10^9^–10^10^ copies g^−1^ DW, whereas *Tn916/1545*, IncQ *oriT* and *intI*2 decreased from 10^9^ to 10^7^, 10^8^ to 10^5^–10^6^, and 10^7^ to 10^6^ copies g^−1^ DW, respectively.

### 3.3. Changes in Bacterial Community During Composting

**Figure 3:**
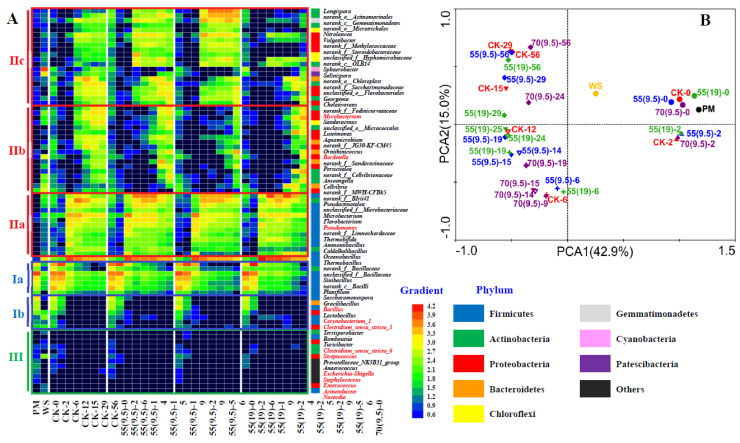
Bacterial community succession over the composting process. (**A**) Heatmap of log_10_-transformed abundances for 60 dominant genera and potential pathogenic genera. The genera are grouped by temporal enrichment patterns: Part Ia and Ib (early-stage predominance), Part IIa–c (later-stage enrichment), and Part III (persistently low abundance). Red letters denote potential pathogens. (**B**) PCA plot showing the variation in bacterial community composition at the OTU level. Black, golden, red, blue, green, and purple symbols represent PM, WS, CK, 55(9.5), 55(19), and 70(9.5), respectively. Symbols ●, ▲, 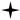, ▽, ▼, and ◆ represent raw, mesophilic, thermophilic, transition, cooling, and maturation samples, respectively.

As shown in Figure 3A and Figure S3, the bacteria of raw compost samples mainly originated from PM because of the similar bacterial communities with an abundance of Firmicutes, especially the genera in Part Ia and Ib of Figure 3A. The dominant phyla were Firmicutes, Actinobacteria, Proteobacteria, and Bacteroidetes (87.28–99.92%) throughout the pig manure composting, which was typical for pig manure composting [31]. However, bacterial communities underwent a great change from Firmicutes, which was the unique dominant phylum (e.g., the genera of Firmicutes in Figure 3A Part Ia) in the earlier composting period, to a prosperity of various phyla (e.g., the genera of Actinobacteria, Proteobacteria, Bacteroidetes, and Chloroflexi in Figure 3A Part IIb and IIc) in the later composting period. Normally, the enrichment of Actinobacteria indicated that compost mixtures trended toward maturity [32].

The genera that decreased during composting (Figure 3A Part Ia and Ib) mainly belonged to Firmicutes. *Clostridium_sensu_stricto_1*, a common anaerobic spore-former in animal gastrointestinal tracts [33], dominated the raw and mesophilic samples (23.4–32.0%) but fell to <0.5% after composting due to aeration. *Lactobacillus*, which mediates organic acidification, rose from 6.9–15.0% (raw) to 47.0–57.4% (mesophilic) before crashing to near-zero under the slight alkalinity of maturation samples. The genera that increased during composting were roughly classified into three types: (1) the genera of Figure 3A Part IIa (mainly Firmicutes) with a low abundance (<0.5%) in raw samples that increased significantly at the beginning of composting, then gradually decreased in the later period at a high abundance after composting; (2) the genera of Figure 3A Part IIb that were significantly enriched after the thermophilic stage for CK, but only significantly increased in the maturation sample for 55(9.5), 55(19), and 70(9.5); (3) the genera of Figure 3A Part IIc that were significantly enriched in the later composting period for CK, 55(9.5), and 55(19), but only increased at the end of composting for 70(9.5). Moreover, the abundance of *Bacillus*, which are strongly resistant to high temperature [34], were enriched dramatically from around 0.5% in raw samples to 41.9–69.9% in thermophilic samples, and then declined to around 8% after composting.

The PCA analysis (Figure 3B) further demonstrated that bacterial communities of each group changed with composting processes, and samples were distributed along a clockwise direction in the graph. Similar to the profiles of ARGs, thermophilic sample 55(19)-19 was clustered with other cooling and maturation samples, while transition sample 70(9.4)-14 and cooling sample 70(9.4)-15 were clustered with other thermophilic samples. These results indicated that: (1) prolonging the thermophilic stage in the 55(19) group did not obstruct the bacterial community from entering into the later period when organic nutrients were insufficient on days 19–24, even though the pile was heated with a higher temperature; and (2) raising the temperature of the thermophilic stage in the 70(9.5) group prevented the bacterial community from entering into the later period even though the pile stopped being heated on days 14–15, which might be due to the fact that the excessive temperature inhibited microbial activity and consumption of organic nutrients. In short, the controls of the thermophilic stage made the earlier and later period of the bacterial community different from the mesophilic–thermophilic and cooling–maturation stage due to the pile temperature.

Pathogenic bacteria carrying ARGs pose serious health and environmental threats. Twenty-five potential pathogenic genera were identified via the VFDB database [35]: eight Firmicutes, seven Proteobacteria, five Actinobacteria, three Epsilonbacteraeota, and one each from Tenericutes and Chlamydiae (Figure 3A). Most remained at trace levels (Figure 3A Part III) or dropped sharply during composting, suggesting that composting effectively reduces pathogens and curtails disease spread. However, *Bacillus*, *Mycobacterium*, and *Bordetella* were significantly enriched during composting, particularly *Bacillus* and *Mycobacterium*, which had high abundances, 7.0–8.6% and 1.5–4.1%, respectively, after composting.

### 3.4. Relationships Between ARGs and Environmental Variables

**Figure 4:**
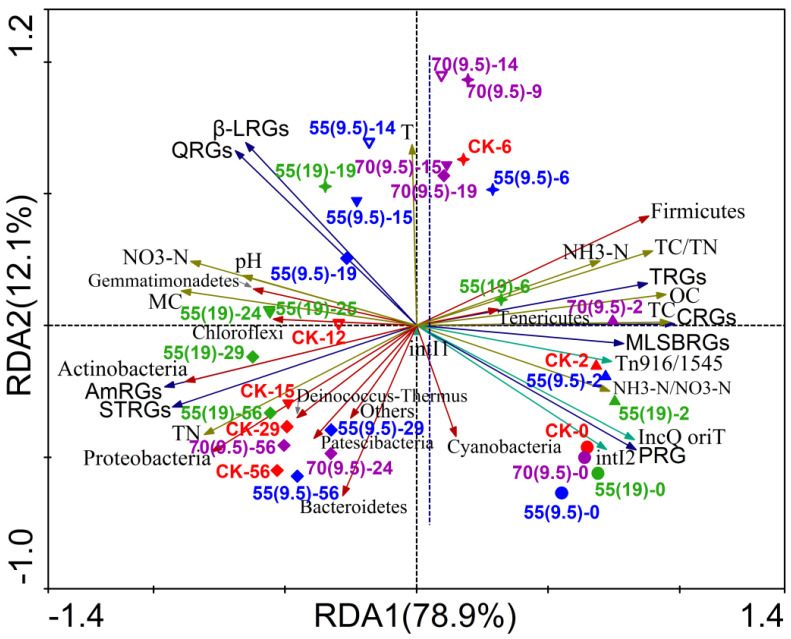
Redundancy analysis (RDA) ordination illustrating the relationships between species variable (ARGs) and environmental variables. Red, blue, green, and purple symbols represent CK, 55(9.5), 55(19), and 70(9.5) samples, respectively. Symbols ●, ▲, 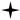, ▽, ▼, and ◆ represent raw, mesophilic, thermophilic, transition, cooling, and maturation samples, respectively. Dark blue, cyan, brown, and red arrows represent ARGs, MGEs, physicochemical factors, and bacterial communities, respectively. Absolute abundance of ARGs and MGEs were calculated in RDA.

The relationships between ARG profiles and environmental variables (MGEs, physicochemical factors and bacterial communities) during composting were elucidated by the RDA analysis. As shown in Figure 4, the explanatory variables accounted for 97.6% of total variation. The first and second axis explained 78.9% and 12.1% of the total variation, respectively. The samples from the earlier and later composting periods were roughly clustered in the right and left parts of the graph, respectively. Early-stage ARG profiles were mainly driven by Firmicutes, MGEs (i.e., *intI*2, Tn*916/1545,* and IncQ *oriT*), and nutrient factors (TC, OC, TN/TC, NH_3_–N/NO_3_^−^–N, and NH_3_–N), which were all positively correlated with PRG, MLSBRGs, CRGs, and TRGs. Later-stage profiles were instead associated with Actinobacteria, Proteobacteria, and several physicochemical factors (e.g., TN and NO_3_^−^–N), which positively correlated to AmRGs and SRTGs. This was in line with the characteristics of the samples in the earlier and later composting periods. Dominant Firmicutes, abundant bioavailable organic matters and NH_3_–N, and the HGT process mediated by Tn*916/1545* led to the characteristic that PRG, MLSBRGs, CRGs, and TRGs were abundant in the earlier composting period, while diverse bacteria and accumulation of TN and NO_3_^−^–N led to the characteristic that AmRGs and SRTGs were abundant in the later composting period.

**Figure 5:**
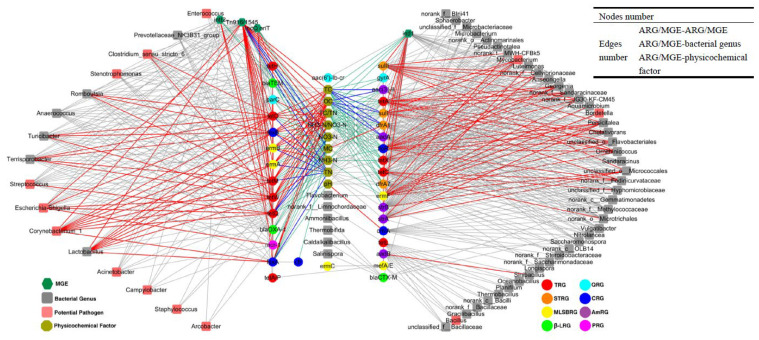
Correlation network of ARGs, MGEs, bacterial genera, and physicochemical factors in 31 compost samples. Absolute abundances were used for ARGs and MGEs. Edge colors: red (positive, *r* ≥ 0.8, *p* < 0.01), grey (positive, 0.6 ≤ *r* < 0.8, *p* < 0.01), green (negative, –0.8 < *r* ≤ –0.6, *p* < 0.01), and blue (negative, *r* ≤ –0.8, *p* < 0.01). Four correlation types are emphasized: ARG–MGE (horizontal gene transfer), ARG/MGE–genus (hosts), ARG/MGE–physicochemical factors, and ARG–ARG (co-occurrence).

Figure 5 and Figure S5 illustrate the complex interrelationships among ARGs, MGEs, bacterial communities, and physicochemical factors across all 31 compost samples. The resulting network was partitioned into two distinct clusters. The right cluster contained 21 enriched ARGs, *intI*1, and 49 bacterial genera; the left cluster grouped 15 decreased ARGs, the other three MGEs, and 16 bacterial genera.

The integron *intI*1, which serves as a proxy for HGT potential and anthropogenic gene contamination [36], exhibited positive correlations with 14 ARGs that were enriched during composting. Notably, several of these—including *aad*A, *sul*I, *tet*A, *flo*R, and *dfr*A1— have been previously documented to co-occur with *intI*1 within various bacterial strains [28,37,38]. It further demonstrated that the process of HGT mediated by *intI*1 promoted the dissemination and enrichment of STRGs and AmRGs in pig manure composting. Transposon Tn*916/1545* and plasmid gene IncQ *oriT* were positively correlated to 14 and 12 decreased ARGs, respectively. Thereafter, conjugative transposons of the Tn*916/1545* family carrying *tet*M and plasmids carrying *bla*_CTX-M_ and *mcr*-1 were reported in some previous studies [39,40]. It indicated that pig manure composting would remove some ARGs (e.g., *tet*M and *mcr*-1) by inhibiting the HGT processes mediated by transposons and plasmids.

ARG–bacteria correlations revealed potential ARG hosts. Most ARGs correlated with multiple genera. For example, t*et*M and *sul*I positively correlated with 10 and 19 genera, respectively, with known hosts including *Clostridium*, *Lactobacillus*, *Streptococcus*, *Anaerococcus* (*tet*M), and *Mycobacterium* (*sul*I) [41,42]. Enriched ARGs (right cluster, Figure 5) averaged 13.9 positive genus correlations, versus 9.7 for decreased ARGs (left cluster), implying that broader host ranges favor ARG enrichment. Conversely, some genera hosted multiple ARGs. *Mycobacterium*, *Streptococcus*, *Anaerococcus*, and eight others positively correlated with 14 ARGs. *Streptococcus*, a pyogenic pathogen, positively correlated with *erm*B, *tet*M, *tet*O, and *tet*W, consistent with known hosts [41,42]. These results indicate diverse ARG hosts and the presence of multiple antibiotic-resistant bacteria (ARB). Notably, 13 potential pathogen genera may host ARGs; *Bacillus*, *Bordetella*, and *Mycobacterium* correlated with three, eight, and 13 enriched ARGs (e.g., *tet*L, *tet*A, *sul*I, and *sul*II), respectively.

The relationships between ARGs and physicochemical factors represented the influence of compost mixture properties on ARGs. Non-nutrition factors (i.e., T, pH, and MC) had fewer or weaker correlations with ARGs, especially T with no correlation with ARGs. On the other hand, nutrition factors had various correlations with ARGs. Spearman correlation analysis further revealed specific association patterns between ARGs and physicochemical factors. Specifically, TN was positively correlated with *tetX*, *floR*, and *strB*, but negatively correlated with *tetW* and *ermB*. NO_3_^−^–N was positively correlated with *sulI* and *aadA*, suggesting that nitrate accumulation in the later stage may indirectly promote the enrichment of certain ARGs by facilitating the proliferation of specific host bacteria (e.g., *Mycobacterium*). In contrast, OC was positively correlated with *tetW* and *ermB*, but negatively correlated with *sulI*. NH_3_–N was positively correlated with *tetM* and *fexA*, but negatively correlated with *sulII*. These results indicate that abundant OC and NH_3_–N in the early stage provided sufficient carbon/nitrogen sources and energy for Firmicutes (e.g., *Clostridium*, *Lactobacillus*), which harbored ARGs such as *tetM* and *ermB*, thereby supporting the persistence of these genes in the early stage. As composting progressed, OC was largely consumed and nitrates gradually accumulated, driving the microbial community shift from Firmicutes to Actinobacteria and Proteobacteria. Host bacteria carrying *sulI* and *aadA* (e.g., *Mycobacterium*) gradually became predominant, leading to a transition of the ARG profile from tetracycline/macrolide dominance in the early stage to sulfonamide/aminoglycoside dominance in the later stage. This interpretation is consistent with the RDA results (OC explaining 82% of ARG variation), further supporting the notion that nutrient composition indirectly shapes ARG profiles by driving community succession.

As shown in Figure S5, a total of 144 positive correlations were identified within the ARG network, of which 51 were classified as strong correlations. Some ARGs shared the same genus correlates. For example, *tet*X, *sul*II, *str*B, and *flo*R were mutually correlated and each positively associated with *Mycobacterium* (a potential pathogenic host). This further confirms the occurrence of multiple ARBs with coexisting ARGs during composting.

## 4. Conclusions

Compared with the spontaneous thermogenic treatment, methods of controlling the thermophilic stage (including prolonging its duration and raising its temperature) substantially decreased the relative abundance of ARGs during pig manure composting. Hence, these methods have positive implications for mitigating the risk of resistance transmission from compost products by decreasing the proportion of ARGs in total bacteria. The composting system harbored active HGT processes, a broad spectrum of ARG hosts, multiple ARBs, and coexisting ARG assemblages. The control of the thermophilic stage affected the variation of OC, implying that nutrients may indirectly shape ARG profiles via bacterial community succession. The positive correlations between *int*I1 and potential pathogens with several enriched ARGs during composting raise concerns about potential health and ecological hazards. Based on the above conclusions, thermophilic stage control strategies alone may not be adequate for effectively reducing ARG risks in pig manure composting. Effective mitigation may instead depend on modifying the nutrient compositon of the compost mixture.

## Figures and Tables

**Table 1:** Compost samples for molecular biology analysis.

	Raw	Mesophilic	Thermophilic	Transition *^a^*	Cooling	Maturation
CK	CK–0 *^b^* [19.6] *^c^*	CK–2 [34.7]	CK–6 [64.6]	CK–12 [49.5]	CK–15 [37.4]	CK–29 [28.1], CK–56 [30.9]
55(9.5)	55(9.5)–0 [19.1]	55(9.5)–2 [38.1]	55(9.5)–6 [65.1]	55(9.5)–14 [57.5]	55(9.5)–15 [30.9]	55(9.5)–19 [28.0], 55(9.5)–29 [33.6], 55(9.5)–56 [29.9]
55(19)	55(19)–0 [18.8]	55(19)–2 [37.4]	55(19)–6 [64.3], 55(19)–19 [54.8],	55(19)–24 [55.0]	55(19)–25 [27.8]	55(19)–29 [24.3], 55(19)–56 [29.9]
70(9.5)	70(9.5)–0 [19.3]	70(9.5)–2 [39.1]	70(9.5)–9 [67.6]	70(9.5)–14 [64.3]	70(9.5)–15 [29.8]	70(9.5)–19 [28.0], 70(9.5)–24 [39.0], 70(9.5)–56 [30.7]

*a* Transition samples were collected at the end of the thermophilic stage or when the water bath was changed to heat preservation through the insulating material. *b* The number in the sample name refers to the sampling time (day). For example, CK-0 is the sample of CK collected at day 0. *c* The number in “[]” refers to the temperature value of the sample.

## Data Availability

The original contributions presented in this study are included in the article/Supplementary Materials. Further inquiries can be directed to the corresponding author.

## Supplementary Materials

The following supporting information can be downloaded at: https://www.mdpi.com/article/10.3390/toxics14070636/s1, Table S1: Categories, mechanisms, primers, thermocycling steps of qPCR and amplification efficiencies in this study; Figure S1: Variations in physicochemical factors during pig manure composting; Figure S2: Variations in relative abundances of ARGs, MGEs and total ARGs during composting; Figure S3: Succession of bacterial communities at phylum level during composting; Figure S4: Hierarchical clustering tree on profiles of ARGs and bacterial communities; Figure S5: Network analysis showing correlations among ARGs and MGEs.
