# Supplementary material for: Insights into the Mechanisms Driving the Dynamics of Antibiotic Resistance Genes During Pig Manure Composting
Source: Toxics. 2026 Jul 21;14(7):636. doi: 10.3390/toxics14070636 (PMC13418462; doi:10.3390/toxics14070636)
Supplement: Supplementary file 1 [file toxics-14-00636-s001.zip › toxics-4401318-supplementary.pdf]

Insights into the mechanisms driving the dynamics of antibiotic resistance genes during pig manure composting

Xun Pan [a], Rukun Cao [b], Mengxue Ge [b], Mengqi Dong [a], Weiwei Ben [b*]

[a] *Solid Waste and Chemicals Management Center, Ministry of Ecology and Environment of the People's Republic of China, Beijing, 100029, China*

[b] *Research Center for Eco-Environmental Sciences, University of Chinese Academy of Sciences, Chinese Academy of Sciences, 18 Shuang-qing Road, Beijing 100085, China*

[*] Corresponding authors.

E-mail address: wwben@rcees.ac.cn (W. Ben)

Captions

Table S1. Categories, mechanisms, primers, thermocycling steps of qPCR and amplification efficiencies in this study.

Figure S1. Variations in physicochemical factors during pig manure composting.

Figure S2. Variations in relative abundances of ARGs, MGEs and total ARGs during composting.

Figure S3. Succession of bacterial communities at phylum level during composting.

Figure S4. Hierarchical clustering tree on profiles of ARGs and bacterial communities.

Figure S5 Network analysis showing correlations among ARGs and MGEs.

References

Table S1. Categories, mechanisms, primers, thermocycling steps of qPCR and amplification efficiencies in this study.

Category	Target Gene	Mechanism	Primer Sequences (5' – 3')	Amplicon Size (bp)	Annealing Temp (°C)	Amplification Efficiency
Tetracyline resistance genes (TRGs)	*tet*A	efflux	F：GCTACATCCTGCTTGCCTTC<br>R：CATAGATCGCCGTGAAGAGG	210	55	102%
	*tet*C	efflux	F：CTTGAGAGCCTTCAACCCAG<br>R：ATGGTCGTCATCTACCTGCC	418	55	94%
	*tet*A/P	efflux	F：CTTGGATTGCGGAAGAAGAG<br>R：ATATGCCCATTTAACCACGC	676	55	99%
	*tet*H	efflux	F：CAGTGAAAATTCACTGGCAAC<br>R：ATCCAAAGTGTGGTTGAGAAT	185	61	92%
	*tet*L	efflux	F：TCGTTAGCGTGCTGTCATTC<br>R：GTATCCCACCAATGTAGCCG	267	55	91%
	*tet*X	inactivation	F：CAATAATTGGTGGTGGACCC<br>R：TTCTTACCTTGGACATCCCG	468	58	101%
	*tet*M	target protection	F：ACAGAAAGCTTATTATATAAC<br>R：TGGCGTGTCTATGATGTTCAC	171	55	101%
	*tet*O	target protection	F：ACGGARAGTTTATTGTATACC<br>R：TGGCGTATCTATAATGTTGAC	171	60	105%
	*tet*Q	target protection	F：AGAATCTGCTGTTTGCCAGTG<br>R：CGGAGTGTCAATGATATTGCA	169	63	103%
	*tet*W	target protection	F：GAGAGCCTGCTATATGCCAGC<br>R：GGGCGTATCCACAATGTTAAC	168	64	107%

Sulfonamide and trimethoprim resistance genes (STRGs)	*sul*I	target replacemen	F：CACCGGAAACATCGCTGCA<br>R：AAGTTCCGCCGCAAGGCT	158	60	108%
	*sul*II	target replacemen	F：CTCCGATGGAGGCCGGTAT<br>R：GGGAATGCCATCTGCCTTGA	190	60	94%
	*dfr*A1	target replacement	F：AGCATTACCCAACCGAAAGT<br>R：TGTCAGCAAGATAGCCAGAT	818	60	100%
	*dfr*A7	target replacement	F：AAATGGCGTAATCGGTAATG<br>R：GTGAACAGTAGACAAATGAAT	324	51	96%
Macrolide, lincosamide, and strepto-gramin B genes (MLSBRGs)	*erm*A	target alteration	F：GAAATYGGRTCAGGAAAAGG<br>R：AAYAGYAAACCYAAAGCTC	332	55	104%
	*erm*B	target alteration	F：GATACCGTTTACGAAATTGG<br>R：GAATCGAGACTTGAGTGTGC	364	58	104%
	*erm*C	target alteration	F：TCAAAACATAATATAGATAAA<br>R：GCTAATATTGTTTAAATCGTCAAT	642	50	95%
	*erm*F	target alteration	F：CGACACAGCTTTGGTTGAAC<br>R：GGACCTACCTCATAGACAAG	309	56	97%
	*mef*A/E	efflux	F：AGTATCATTAATCACTAGTGC<br>R：TTCTTCTGGTACTAAAAGTGG	348	45	96%
β-lactam resistance genes (β-LRGs)	*bla*$_{TEM}$	inactivation	F：ATCAGCAATAAACCAGC<br>R：CCCCGAAGAACGTTTTC	516	60	108%
	bla$_{CTX-M}$	inactivation	F：ATGTGCAGYACCAGTAARGTKATGGC<br>R：ATCACKCGGRTCGCCNGGRAT	300	60	95%
	bla$_{OXA-1}$	inactivation	F：ATATCTCTACTGTTGCATCTCC<br>R：AAACCCTTCAAACCATCC	619	55	93%

Quinolone resistance genes (QRGs)	*aac(6')-Ib-cr*	inactivation	F：TTGCGATGCTCTATGAGTGGCTA<br>R：CTCGAATGCCTGGCGTGTTT	482	58	97%
	*gyr*A	target alteration	F：GAYGGNYTNAARCCNGTNCA<br>R：GCCATNCCNACNGCDATNCC	421	48	91%
	*par*C	target alteration	F：GCGAATAAGTTGAGGAATCAG<br>R：AGCTCGGAATATTTCGACAAC	418	55	99%
Chloram-phenicol resistance genes (CRGs)	*cfr*	target alteration	F：TGTGCTACAGGCAACATTGGAT<br>R：CAAATACTTGACGGTTGGCTAGAG	148	55	105%
	*cml*A	efflux	F：GCCAGCAGTGCCGTTTAT<br>R：GGCCACCTCCCAGTAGAA	158	55	105%
	*fex*A	efflux	F：ATTCTCCCGCAAATAACG<br>R：TCGGCTCAGTAGCATCACG	156	52	104%
	*fex*B	efflux	F：CCCGATAGATAATAATACAG<br>R：CACCAATAGTGGGAAGAT	134	45	102%
	*flo*R	efflux	F：CGGTCGGTATTGTCTTCACG<br>R：TCACGGGCCACGCTGTAT	171	56	97%
Aminogly-coside resistance genes (AmRGs)	*aac(3)-II*	inactivation	F：TGAAACGCTGACGGAGCCTC<br>R：GTCGAACAGGTAGCACTGAG	370	55	107%
	*str*A	inactivation	F：GCTAACGCCGAAGAGAACTG<br>R：AGGTGTCCGCAATGAGAACA	207	56	105%
	*str*B	inactivation	F：CGGCTGGCTGGTGATAGAT<br>R：GCGTTGCTCCTCTTCTCCA	238	60	105%
	*aad*A	inactivation	F：AAATTCTTCCAACTGATCTGCG<br>R：CCTGAACAGGATCTATTTGAGGC	276	58	107%

	*aad*B	inactivation	F：TGGTGGTACTTCATCGGCATA<br>R：GTTACTTGACTGCGAACCTGCT	175	58	106 %
Polymyxin resistance gene (PRG)	*mcr*-1	target alteration	F：TCCAAAATGCCCTACAGACC<br>R：GCCACCACAGGCAGTAAAAT	205	56	106%
Mobile genetic elements (MGEs)	*intI*1	Class I integron	F：GGCTTCGTGATGCCTGCTT<br>R：CATTCCTGGCCGTGGTTCT	146	55	100%
	*intI*2	Class II integron	F：GTTATTTTATTGCTGGGATTAGGC<br>R：TTTTACGCTGCTGTATGGTGC	164	55	105%
	Tn*916 /1545*	transposon	F：GACAGTATTAAGCCATCAGAC<br>R：TCTTCCGAACACAATCATCT	142	50	106%
	IncQ *oriT*	Conjugative plasmids	F：TTCGCGCTCGTTGTTCTTCGAGC<br>R：GCCGTTAGGCCAGTTTCTCG	191	62	93%
	16S rRNA	biomass	F：CCTACGGGAGGCAGCAG<br>R：TTACCGCGGCTGCTGGCAC	193	60	101%

1) R=A,G; Y=C, T; K=G, T; S=G, C; M=A, C; W=A, T; B=G, C, T; V=A, G ,C; D=A, G, T; H=A, C, T; N=A, G, C, T.

2) The thermocycling steps of qPCR: (1) 95 ºC, 5 min; (2) 95 ºC, 15 s; (3) annealing temperature, 30 s; (4) 72 ºC, 30 s; (5) plate read; (6) repeat steps (2) trough (5) 39 more times; (7) melt-curve analysis, 60 ºC − 95 ºC, 0.2 ºC read.

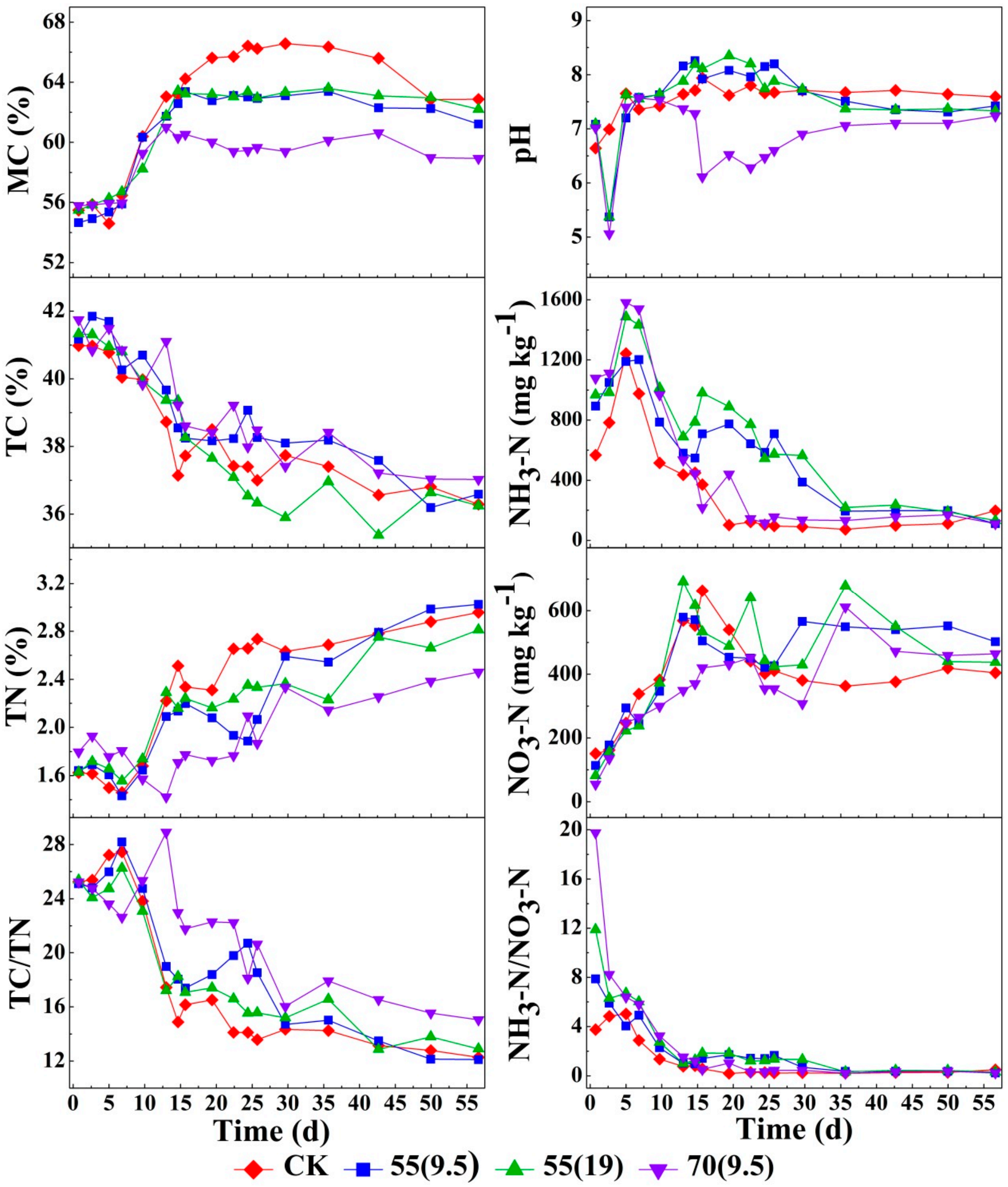

Figure S1. Variations in physicochemical factors during pig manure composting. MC: moisture content. TC: total carbon, by dry weight. TN: Total nitrogen, by dry weight. NH_3-N: dissolvable NH_3-N, by fresh weight. NO_3^--N: dissolvable NO_3^--N, by fresh weight.

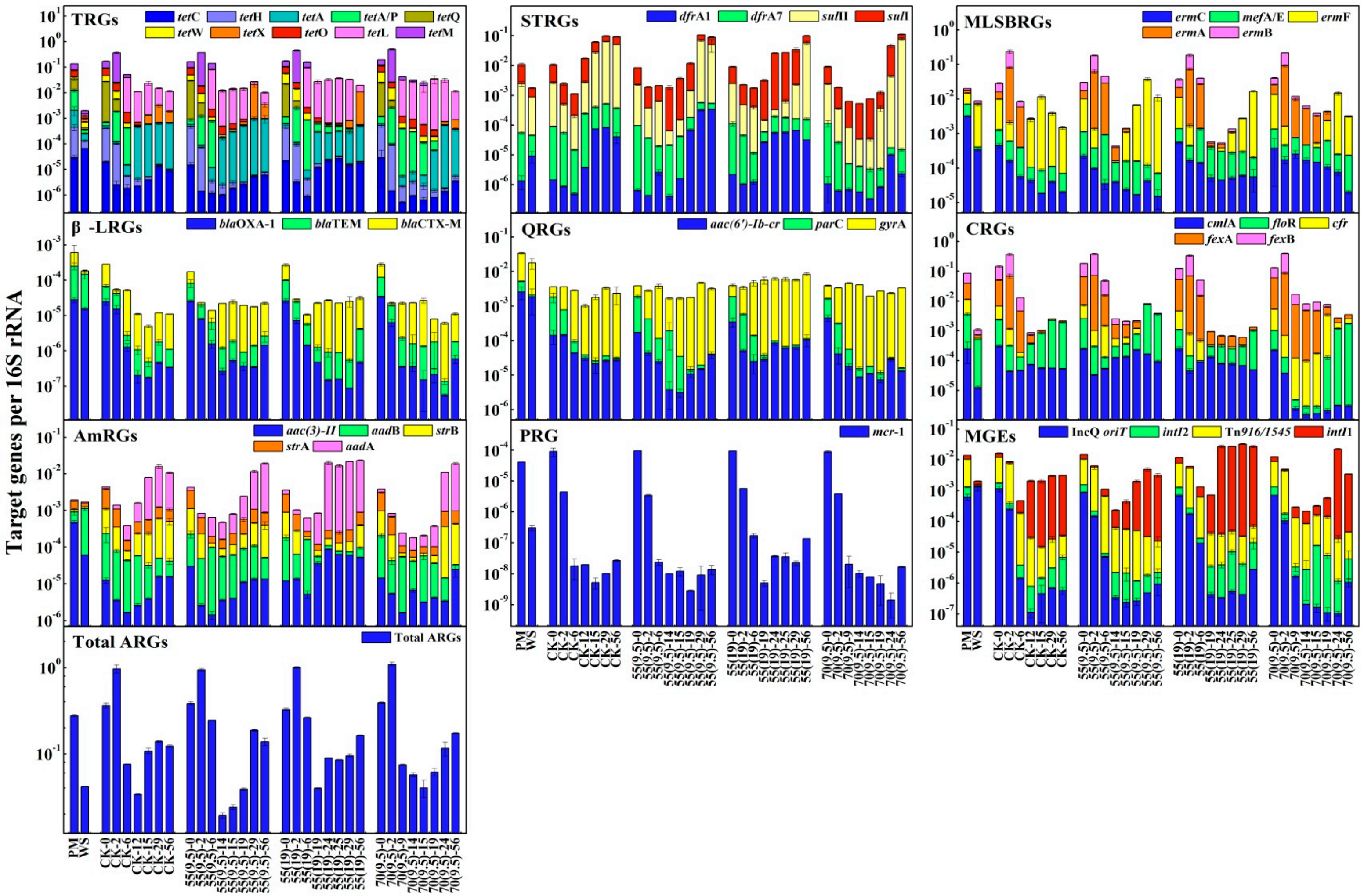

Figure S2. Variations in relative abundances of ARGs, MGEs and total ARGs during composting.

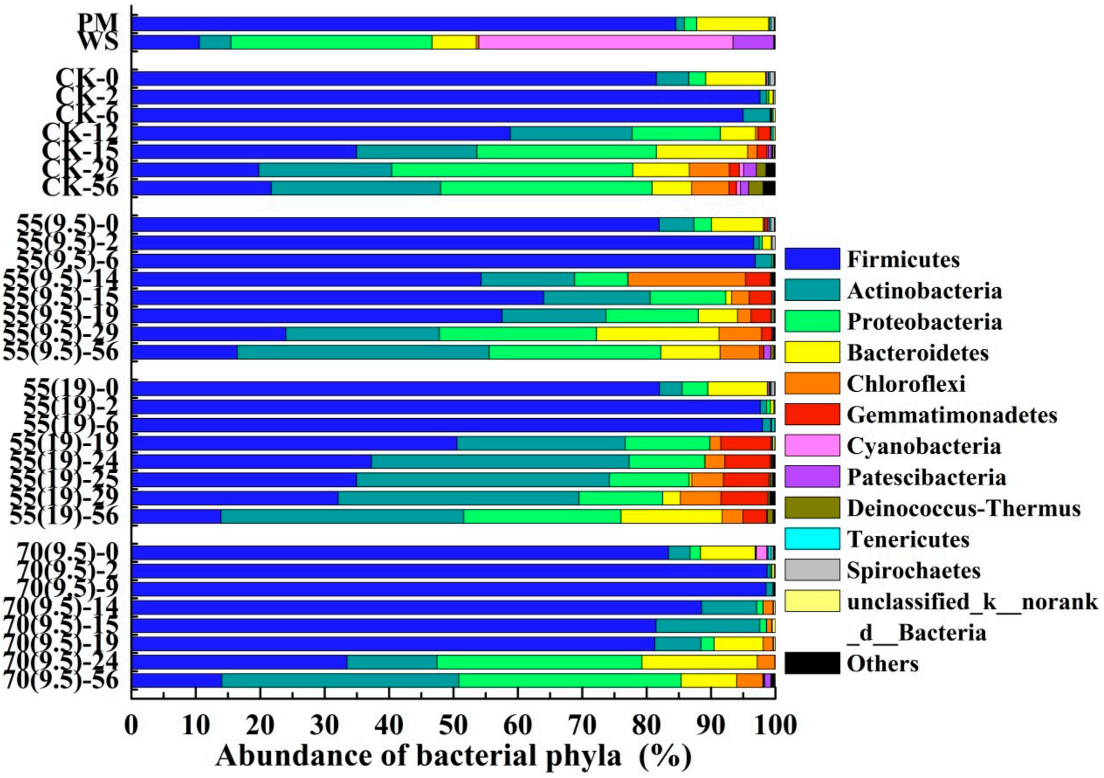

Figure S3. Succession of bacterial communities at phylum level during composting.

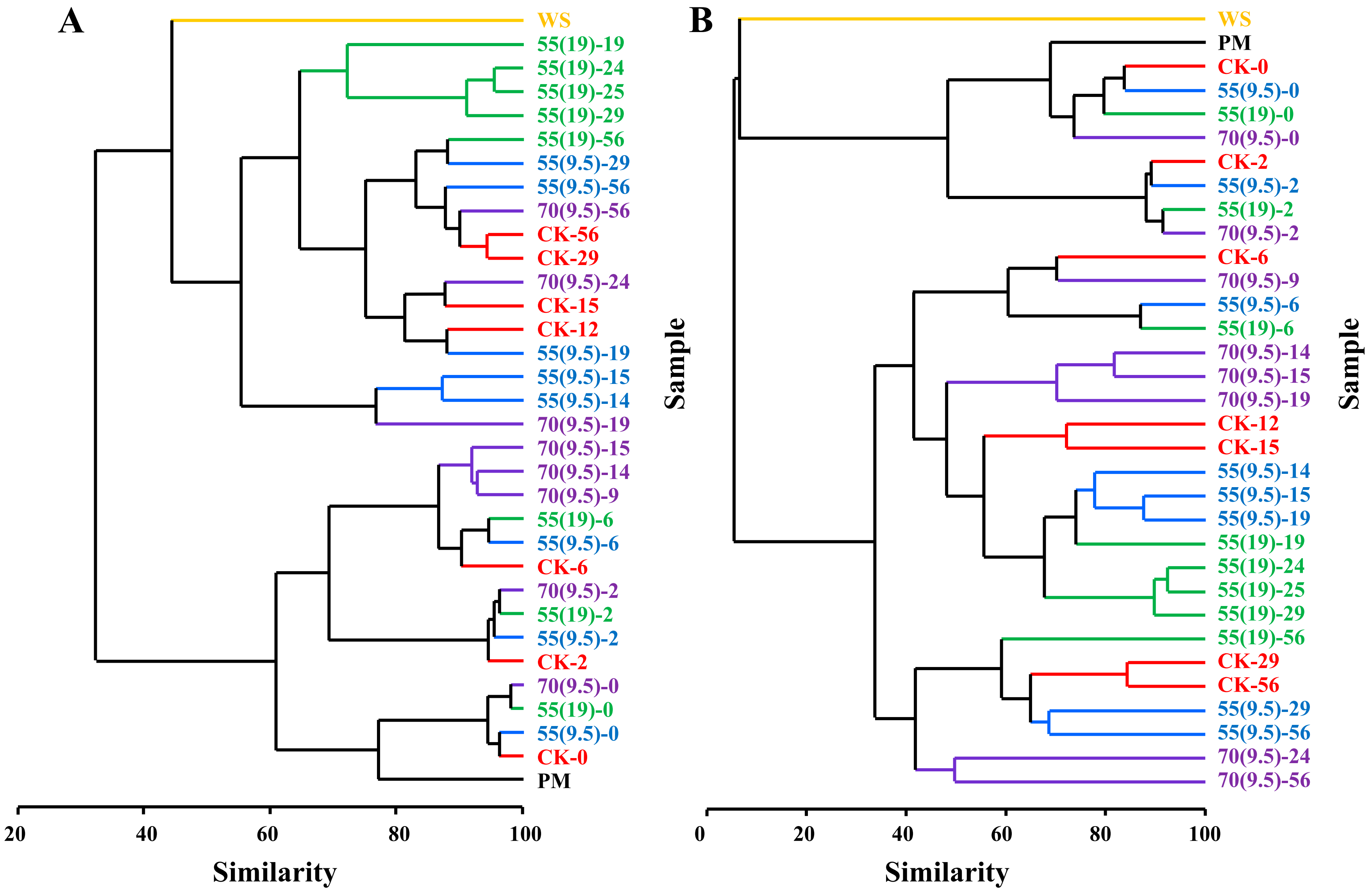

Figure S4. Hierarchical clustering tree on profiles of ARGs (A) and bacterial communities (B). Profiles of ARGs were evaluated using absolute abundance. Bacterial communities were at OTU level.

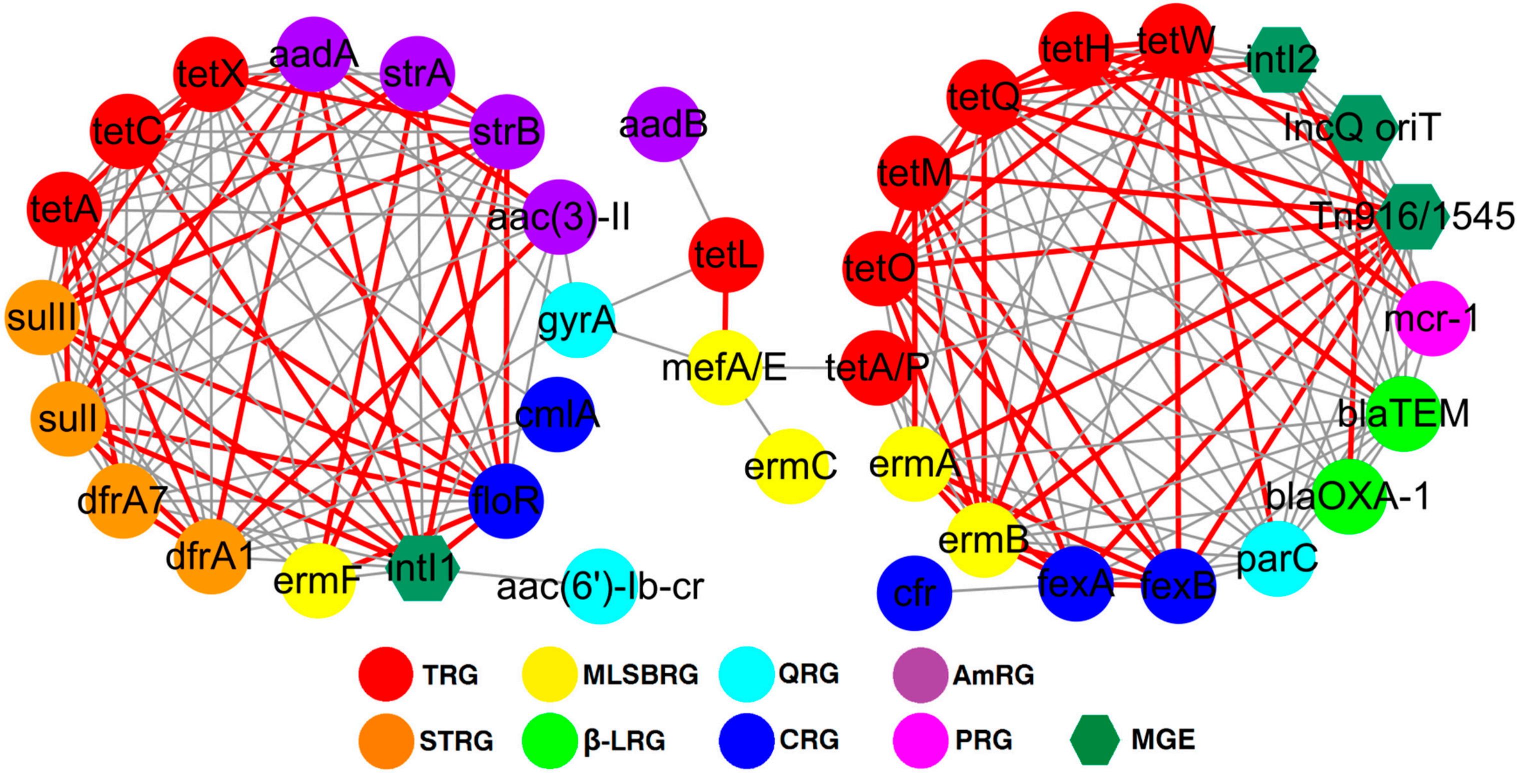

Figure S5. Network analysis showing correlations among ARGs and MGEs. Red and grey edges represented significantly positive ($r \geq 0.8$, $p < 0.01$) and positive ($0.6 \leq r \leq 0.8$, $p < 0.01$) correlations, respectively. Positive correlations between ARG and ARG, and between ARG and MGE were used to demonstrated the co-occurrence of ARGs and the horizontal gene transfer, respective
